# Highly porous scaffolds of PEDOT:PSS for bone tissue engineering

**DOI:** 10.1016/j.actbio.2017.08.045

**Published:** 2017-10-15

**Authors:** Anne Géraldine Guex, Jennifer L. Puetzer, Astrid Armgarth, Elena Littmann, Eleni Stavrinidou, Emmanuel P. Giannelis, George G. Malliaras, Molly M. Stevens

**Affiliations:** a Department of Materials, Department of Bioengineering and Institute of Biomedical Engineering, Imperial College London, Prince Consort Road, London SW7 2AZ, United Kingdom; b National Lung and Heart Institute, Imperial College London, 435 Du Cane Road, London W12 0NN, United Kingdom; c Materials Science and Engineering, College of Engineering, Cornell University, 176 Kimball Hall, Ithaca, NY 14853, United States; d Department of Bioelectronics, Ecole Nationale Supérieure des Mines de Saint Etienne, Centre Microélectronique de Provence, 880 Route de Mimet, 13541 Gardanne, France

**Keywords:** Bone tissue engineering, PEDOT:PSS, Conductive scaffolds, Porosity

## Abstract

Conjugated polymers have been increasingly considered for the design of conductive materials in the field of regenerative medicine. However, optimal scaffold properties addressing the complexity of the desired tissue still need to be developed. The focus of this study lies in the development and evaluation of a conductive scaffold for bone tissue engineering. In this study PEDOT:PSS scaffolds were designed and evaluated *in vitro* using MC3T3-E1 osteogenic precursor cells, and the cells were assessed for distinct differentiation stages and the expression of an osteogenic phenotype.

Ice-templated PEDOT:PSS scaffolds presented high pore interconnectivity with a median pore diameter of 53.6 ± 5.9 µm and a total pore surface area of 7.72 ± 1.7 m^2^·g^−1^. The electrical conductivity, based on I-V curves, was measured to be 140 µS·cm^−1^ with a reduced, but stable conductivity of 6.1 µS·cm^−1^ after 28 days in cell culture media. MC3T3-E1 gene expression levels of *ALPL, COL1A1* and *RUNX2* were significantly enhanced after 4 weeks, in line with increased extracellular matrix mineralisation, and osteocalcin deposition. These results demonstrate that a porous material, based purely on PEDOT:PSS, is suitable as a scaffold for bone tissue engineering and thus represents a promising candidate for regenerative medicine.

**Statement of Significance:**

Tissue engineering approaches have been increasingly considered for the repair of non-union fractions, craniofacial reconstruction or large bone defect replacements. The design of complex biomaterials and successful engineering of 3-dimensional tissue constructs is of paramount importance to meet this clinical need. Conductive scaffolds, based on conjugated polymers, present interesting candidates to address the piezoelectric properties of bone tissue and to induce enhanced osteogenesis upon implantation. However, conductive scaffolds have not been investigated *in vitro* in great measure. To this end, we have developed a highly porous, electrically conductive scaffold based on PEDOT:PSS, and provide evidence that this purely synthetic material is a promising candidate for bone tissue engineering.

## Graphical abstract

**Figure:**
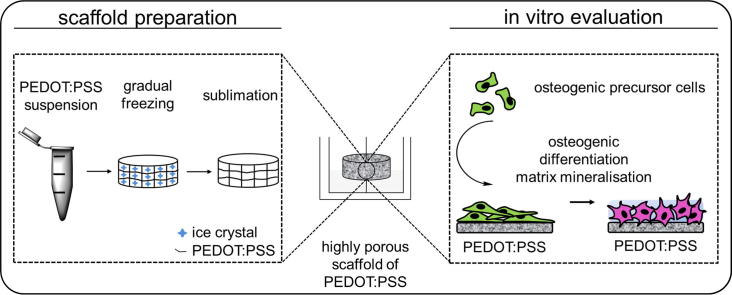

## 1 Introduction

Conjugated polymers, in particular poly(3,4-ethylenedioxythiophene) polystyrene sulfonate (PEDOT:PSS), have been numerously reported as potential candidates for biomedical applications and it has been hypothesised that they would present an ideal substrate for the growth and electrical stimulation of various cell types, most prominently osteogenic cells [1]. Actual long term *in vitro* studies and evaluations of PEDOT:PSS as osteoinductive scaffolds are far fewer than the amount of published studies on material characterisation in other fields [2]. To the best of our knowledge, this is the first report of the differentiation of osteogenic precursor cells into mature, mineralised osteoblasts on a porous PEDOT:PSS scaffold.

Conductive or conjugated polymers have found wide application in multiple fields, including photovoltaics, optoelectronics, biosensors, and regenerative medicine [3], [4], [5], [6]. Polypyrrole (PPy), polyaniline (PANi), or PEDOT, among other polythiophene derivatives, are of utility as electroactive substrates or scaffolds for tissue engineering applications [7], [8]. The intrinsic conductivity of these conjugated systems is, however, as low as 10^−7^ to 10^−11^ S·cm^−1^ and it is the process of doping that converts these materials into conductors. Controlling the size, charge, and stability of the counter ions is of paramount importance to control and fine-tune the electrochemical properties of the material and this has a major effect on the *in vitro* or *in vivo* performance of such conductive scaffolds. In this context, the ionomer mixture of PEDOT:PSS is particularly interesting due to its stability, cytocompatibility and conductivity. The electrical properties can be further improved by solvent treatment or the addition of secondary dopants, rendering this system highly versatile for research purposes [9], [10], [11], [12]. PEDOT:PSS dispersions are furthermore commercially available and present high processability. Recent studies by Shahini *et al.*, and Wan *et al.*, reported on an ice-templating method to process dispersions of PEDOT:PSS into porous, three dimensional scaffolds, allowing for their use in tissue engineering [1], [13].

Large-scale bone defects, such as non-healing fractures, bone tumour ablations, bullet wounds, craniofacial surgery and reconstruction, all occur at high prevalence, and impose significant physical and psychological discomfort to patients. Countless research efforts target the regeneration of existing bone or replacement of lost bone by complex biomaterials and tissue engineering approaches and the implantation of *ex vivo* generated bone tissue [14], [15]. However, advancements in bone union and regeneration of large defects are still needed [16], [17], [18].

In 1957, Fukada and Yasuda reported that bone has inherent piezoelectric properties, suggesting that electrical stimulation would enhance the fusion of non-union bone fractures or increase healing rates [19], [20]. Based on clinical trials, or *in vitro* evaluations, no consensus on the required field strength, wave-form, amplitude or duration could be reached [21], [22], but there is growing evidence that external electrical stimulation has an effect on cell proliferation, migration, and mineralisation [23], [24]. Conductive materials may even further support osteogenesis *in vitro* and bone regeneration *in vivo* upon biomaterial implantation [25], [26]. In this respect, PEDOT:PSS scaffolds provide an interesting cell culture substrate for osteogenic differentiation.

*In vitro* evaluations with human mesenchymal stem cells or mouse pre-adipocytes confirmed cell adhesion and viability on porous, freeze dried PEDOT:PSS scaffolds [1], [13]. Recent studies further reported on the growth of human foetal mesenchymal stem cells on 3D-printed macroporous polycaprolactone (PCL) scaffolds, surface coated with PEDOT [27]. In depth cell analysis, including osteogenic differentiation after prolonged culture period, is however lacking in all studies and have, to our knowledge, not been reported elsewhere for a 3D PEDOT:PSS material. It is therefore timely to first evaluate the response and differentiation of pre-osteoblasts on conducting PEDOT:PSS scaffolds to gain important insight into their cell material interactions and osteoinductive potential. To this end, the current study focused on the development and evaluation of a highly porous, conductive scaffold for bone tissue engineering. We hypothesised that porous PEDOT:PSS scaffolds are suitable and would support the differentiation of osteogenic precursor cells (MC3T3-E1). The effect of an electrically conductive scaffold on osteogenic differentiation (without applying external electrical stimulus) has not been addressed to date and it remains open to what extent such a 3D scaffold will find clinical applicability. Here, we provide evidence for increased gene expression levels of the osteogenic markers *ALPL, COL1A1,* and *RUNX2*, in line with matrix mineralisation and hydroxyapatite nodule formation, osteocalcin deposition and alkaline phosphatase activity – which are all indicative of the versatility of PEDOT:PSS as a scaffold material for bone tissue engineering.

## 2 Materials and methods

### 2.1 Scaffold preparation and characterisation

#### 2.1.1 Ice templating

Scaffolds were prepared from a PEDOT:PSS dispersion (1.25 wt% in water, Ossila Ltd, Sheffield, UK), supplemented with 0.25 v/v% 4-dodecylbenzenesulfonic acid (DBSA, Sigma, St. Louis, MO, USA) as a secondary dopant and 0, 1, 2 or 3 v/v% 3-glycidoxypropyltrimethoxysilane (GOPS, Sigma, St. Louis, MO, USA) as a crosslinker. The dispersion was filled into 2 mL cryogenic vials (VWR, Radnor, PA, USA), placed in a coolcell box (VWR, Radnor, PA, USA) and frozen in a −26 °C or −80 °C freezer overnight. Specified by the manufacturer, the coolcell box ensures a freezing rate of −1 °C·min^−1^ when placed at -80 °C, whereas a slower freezing rate is expected when placed at −26 °C due to the smaller temperature difference (ΔT). After gradual freezing, all tubes were placed at −80 °C overnight prior to freeze drying. Lyophilisation was accomplished on a Heto, PowerDry LL1500 (Thermo Electron Cooperation, Waltham, MA, USA) for at least 8 hours. Scaffolds were then annealed at 140 °C for 2 h. For all further experiments, PEDOT:PSS samples were cut into disks of 6 mm diameter and 1 to 2 mm thickness.

#### 2.1.2 Scaffold morphology and pore size characterisation

PEDOT:PSS discs were gold or chromium sputtered and imaged using scanning electron microscopy (JSM6010 LA) at an acceleration voltage of 20 kV and a spot size of 50. Pore diameters were measured with ImageJ (http://imagej.nih.gov/ij/). Total pore surface area, median and mean pore diameter were further assessed by automatic mercury intrusion porosimetry (PoreMaster33, Quantachrome Instruments, Hook, UK). PEDOT:PSS cylinders of known weight were placed in a mercury penetrometer that was evacuated, and then filled with mercury at a gradual increase to 50 psi. Average pore diameter on SEM images was calculated as:

(1) $$d=\sqrt{\left(l\times w\right)}$$

based on 100 individual pores per scaffold (n = 3).

Total pore surface area was calculated as:

(2) $$S=\frac{1}{\gamma |\text{cos}(\theta )|}\int_{0}^{V_{\mathrm{tot}}}\mathrm{pdV}$$

where *γ* = 0.480 N·m^−1^ is the surface tension and *θ* = 140° is the contact angle of mercury, *p* the pressure and *V* the total volume of intruded mercury. Total surface was normalised to scaffold weight and presented as m^2^·g^−1^. Mean pore diameter was calculated as:

(3) $$d_{\mathrm{mean}}=4\frac{V_{\mathrm{tot}}}{S}$$

whereas median pore diameter is the diameter at 50% total volume intrusion. Porosity measurements were assessed for n = 5 individual replicates (scaffolds) for the condition used in cell studies (2% GOPS, −26 °C) and n = 1 for all other samples.

#### 2.1.3 Mechanical properties

Mechanical properties were assessed in unconfined compression using an ElectroForce 3200 System (Bose, Eden Prairie, MN, USA) equipped with a 22 N load cell. Dry and wet (incubated in PBS overnight) 6 mm diameter scaffolds were pre-loaded to 0.03 N, then loaded to 40% compression at a strain rate of 0.1% s^−1^, assuming quasi-static loading. The compressive modulus was calculated based on the slope of the linear regression of the stress-strain curve up to 30% strain. Analysis was accomplished in N = 4 individual experiments with three replicates each, resulting in n = 12 scaffolds.

#### 2.1.4 Electrical properties

Electrical conductivity was assessed at three different time points. Scaffold conductivity was measured on pristine, as produced samples, on samples incubated in pure foetal bovine serum (FBS, Gibco, Life technologies) and on samples incubated in culture media for four weeks. The additional time points represent pre cell seeding (after preconditioning in serum) and 28 days post seeding, and give an indication on the doping and electrical stability of PEDOT:PSS scaffolds. Control groups consisted of scaffolds prepared without the secondary dopant DBSA. FBS or media incubated scaffolds were rinsed in deionised water and air dried prior to measurements. Scaffolds of 6 mm in diameter were placed between two rectangular copper electrodes coated with Gallium-Indium eutectic (Ga/In, Sigma, St. Louis, MO, USA). The I-V characteristics were measured using a Keithley 2400 and recorded using customised LabVIEW software. Output current was measured at set voltage from 0 to 1 V and at reverse bias with 0.01 V intervals. Electrical conductivity σ=1/ρ was calculated based on the slope of the linear regression in the ohmic region (resistance R) and resultant resistivity ρ=R∗(A/d), where *d* is the thickness of the scaffold (measured for each scaffold with a vernier caliper) and *A* *=* *0.1 cm^2^* is the surface area of the electrodes. I-V curves were acquired on n = 3 scaffolds per condition.

#### 2.1.5 Calcium accumulation on scaffolds

Calcium accumulation on materials has been identified as a potential factor to act as nucleation for osteogenic mineralisation [28]. Therefore, the potential of calcium accumulation of PEDOT:PSS scaffolds was assessed by incubating cell-free scaffolds in culture media. Specifically, four different conditions were chosen: dry, as produced scaffolds (ctrl), serum incubated scaffolds (d4 FBS), and scaffolds incubated in proliferation media for 1 or 7 days (d1, d7) followed by differentiation media for 21 days, to mirror cell experiments (d28).

PEDOT:PSS scaffolds were assessed for mineral deposition based on Ca^2+^ complexation with o-CPC. Scaffolds were harvested, rinsed in PBS and frozen in 0.1 v/v% triton-X at −20 °C.

After three freezing / thawing cycles, scaffolds were incubated in 0.75 M acetic acid and 0.1 v/v% triton-X supernatant at a 1:1 ratio for 6 h. The acidic solution with dissolved calcium was then incubated with a 0.01 w/v% o-cresolphtalein complexone solution (o-CPC, Sigma, St. Louis, MA, USA) in 0.25 M sodium borate buffer (pH 10, Sigma, St. Louis, MA, USA). Absorbance was measured at 570 nm (Perkin Elmer EnSpire Plate Reader, Waltham, MA, USA) and quantified with a Ca^2+^ standard curve from 1 M CaCl_2_ solution (Sigma, St. Louis, MA, USA). Evaluation was accomplished in N = 3 individual experiments with three replicates each, resulting in n = 9 scaffolds.

### 2.2 *In vitro* evaluation

#### 2.2.1 Cell expansion and culture

Cell culture plates were prepared as previously reported [29]. In brief, 48-well plates were filled with polydimethylsiloxane (PDMS, SYLGARD® 184 Elastomer Kit, VWR, Radnor, PA, USA). PEDOT:PSS scaffolds of 6 mm diameter and 1 to 2 mm thickness were blanked with biopsy punches (Miltex, Integra Life Sciences, Hampshire, UK) and placed on cured PDMS. Scaffolds were fixed with 0.15 mm insect pins (Watkins and Doncaster, Leominster, UK) and sterilised under UV overnight. Scaffolds were preconditioned prior to cell seeding in foetal bovine serum (FBS, Invitrogen, Life Technologies, Carlsbad, CA, USA) supplemented with 50 ng·mL^−1^ penicillin/streptomycin (Invitrogen, Life Technologies, Carlsbad, CA, USA) for 48 hours, followed by 5 ng·mL^−1^ penicillin/streptomycin for 48 hours.

Mouse pre-osteogenic precursor cells (MC3T3-E1, ATCC, Middlesex, UK) were expanded in α-MEM supplemented with 10 v/v% FBS and 5 ng·mL^−1^ penicillin/streptomycin. MC3T3-E1 were seeded at a density of 250,000 cells per scaffold (0.9·10^−6^ cells·cm^−2^) according to previously published protocols [29] and cultured for 7 days in basal media. Osteogenic differentiation was initiated by α-MEM supplemented with 10 v/v% HyClone FBS (Thermo Fisher Scientific, Loughborough, UK), 5 ng·mL^−1^ penicillin/streptomycin, 5 µg·mL^−1^ L-ascorbic acid (Sigma) and 2 mM β-glycerophosphate disodium salt (Sigma). Media was changed every second day. MC3T3-E1 were cultured in differentiation media for 21 days, resulting in a total culture time of 28 days. *In vitro* evaluation was accomplished with N = 4 individual experiments with three replicates each, resulting in n = 12 scaffolds.

#### 2.2.2 DNA quantification and alkaline phosphatase activity (ALP)

Cell proliferation was assessed based on DNA quantification at four different time points (day 0, day 1, day 7 and day 28) according to previously published protocols [30]. Day 0 refers to an aliquot of the initial cell seeding suspension (250,000 cells, see 2.2.1).

Briefly, constructs were washed in 1x PBS, and transferred to 1.5 mL Eppendorf tubes. Cells were lysed in 0.1 v/v% triton-X and repeated freeze thawing cycles. DNA was quantified with Hoechst and based on a standard curve of calf thymus DNA (Sigma). Fluorescence was measured at 360 nm excitation and 460 nm emission wavelengths (Perkin Elmer EnSpire Plate Reader, Waltham, MA, USA). ALP activity on day 0 aliquots, and on day 1, day 7 and day 28 constructs was quantified based on the dephosphorylation of p-nitrophenyl phosphate (pNPP, Sigma) by ALP. Cell lysate was incubated with pNPP at 37 °C for 2 h. Serial dilutions of paranitrophenol served as a standard curve for quantification. Absorbance was measured at 405 nm (Perkin Elmer EnSpire Plate Reader, Waltham, MA, USA). Enzymatic activity was then calculated based on the conversion rate of 1 U = 1 µmol pNPP per min.

#### 2.2.3 Cell morphology and mineralisation

Cell morphology and mineralisation were visualised by scanning electron microscopy. Substrates were fixed in 3.7 v/v% formaldehyde solution and dehydrated in an ascending series of ethanol. Substrates were then dried in hexamethyldisiloxane (HMDS, Sigma) and placed under vacuum. Prior to image acquisition, substrates were gold or chromium sputtered. Images of low magnification were acquired on a JSM6010 LA at 15 kV acceleration and 50SS, images of high magnification on a LEO Gemini 1525 FEGSEM at 20 kV acceleration voltage, equipped with a detector for backscattered electrons. Energy dispersive X-ray spectroscopy (EDX) was recorded after imaging and analysed with Inca software (Oxford Instruments, High Wycombe, UK). Calcium deposition and Ca^2+^ quantification of cells on scaffolds were assessed based on o-CPC complexation, as described above under 2.1.5.

#### 2.2.4 Osteogenic differentiation

Osteogenic differentiation of pre-osteogenic precursor cells was assessed after 28 days in culture. Gene expression, matrix mineralisation and osteocalcin deposition was compared to samples harvested at day 1 and day 7 post seeding. Onset of osteogenic differentiation was assessed by qPCR of early osteogenic markers *ALP* and *RUNX2*, while *COL1A1* gene expression was also assessed. Furthermore, osteogenic maturation and specifically extracellular matrix deposition and hydroxyapatite formation, were assessed based on histological sections stained for osteocalcin and via OsteoImage staining, respectively.

For qPCR, scaffolds were washed in 1x PBS and lysed in RTL buffer, supplemented with 1 v/v% β-mercaptoethanol. RNA was isolated with an RNA isolation kit (RNeasy Mini Kit, Qiagen, Hilden, Germany), RNA concentration measured with NanoDrop Spectrometer (Thermo Fisher Scientific, Perth, UK). cDNA synthesis was accomplished with a reverse transcriptase kit (Reverse Transcriptase Kit, Qiagen, Hilden, Germany), following manufacturer’s instructions. Samples were analysed for the expression of *ALPL*, *COL1A1* and *RUNX2*, normalised to the housekeeping gene *18S* (all Invitrogen). Sequences of forward and reverse primer are found in Table 1. qPCR was run on a QuantStudio6 real-time PCR System (Applied Biosystems, Foster City, CA, USA). Samples were run with Express SYBR green super mix (Invitrogen). Fold expression was calculated as 2^−ΔΔCt^ normalised to 18S and gene expression on day 0.

**Table 1:** Forward and reverse primers utilised in the study.

	Forward	Reverse
*18S*	5′-GTAACCCGTTGAACC-3′	5′CCATCCAATCGGTAGTAGTAGCG-3′
*ALPL*	5′-GATCGGGACTGGTACTCGGATAA-3′	5′CACATCAGTTCTGTTCTTCGGGTAC-3′
*COL1A1*	5′-TTCTCCTGGTAAAGATGGTGC-3′	5′GGACCAGCATCACCTTTAACA-3′
*RUNX2*	5′-CCGCCTCAGTGATTTAGGGC-3′	5′GGGTCTGTAATCTGACTCTGTCC-3′

Hydroxyapatite formation was characterised with OsteoImage (Lonza, Slough, UK). Following the manufacturers instruction, cells were washed in wash buffer, followed by incubation with OsteoImage staining agent. Nuclei were counterstained with DAPI (Sigma).

Immunohistochemistry was accomplished on formaldehyde fixed samples. Samples were incubated with anti-osteocalcin antibody (rabbit anti mouse, aa 59–74, MERCK, Millipore, Darmstadt, Germany) followed by a fluorescently labelled secondary antibody (anti-rabbit IgG, Alexa 555, Invitrogen, Life technologies, Carlsbad, CA, USA). Constructs were then embedded in O.C.T (Leica Biosystems GmbH, Nussloch, Germany) and cryosectioned on a cryostat (Model OT, Bright Instruments, Huntington, UK) at −25 °C specimen temperature and 10 µm slice thickness. Slides were mounted with glycergel mounting media (Dako, Agilent Technologies, Ely, UK). Images were acquired on a confocal microscope (Leica SP5 MP inverted (SAFB 408), Wetzlar, Germany).

### 2.3 Statistical analysis

Results are presented as mean ± SD. Significant differences were assessed by nonparametric Kruskal-Wallis test followed by pairwise post-hoc analysis with a Mann-Whitney test. Results were accepted as significantly different for *p* < 0.05. Four independent experiments with three replicates each (cell-scaffold samples) were accomplished.

## 3 Results

### 3.1 Scaffold development and characterisation

#### 3.1.1 Scaffold morphology

**Fig. 1:**
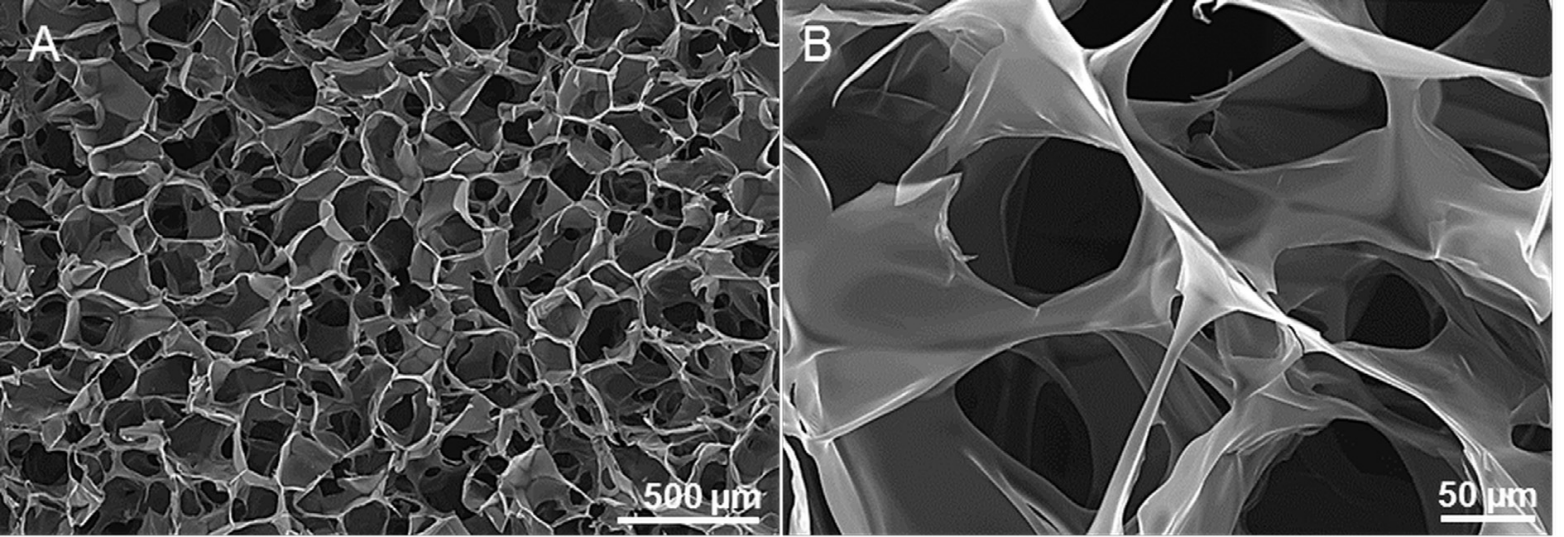
A) and B) Scanning electron microscopy (SEM) images of PEDOT:PSS scaffolds. The highly interconnected, porous structure was obtained by ice templating and subsequent sublimation of a PEDOT:PSS dispersion.

**Fig. 2:**
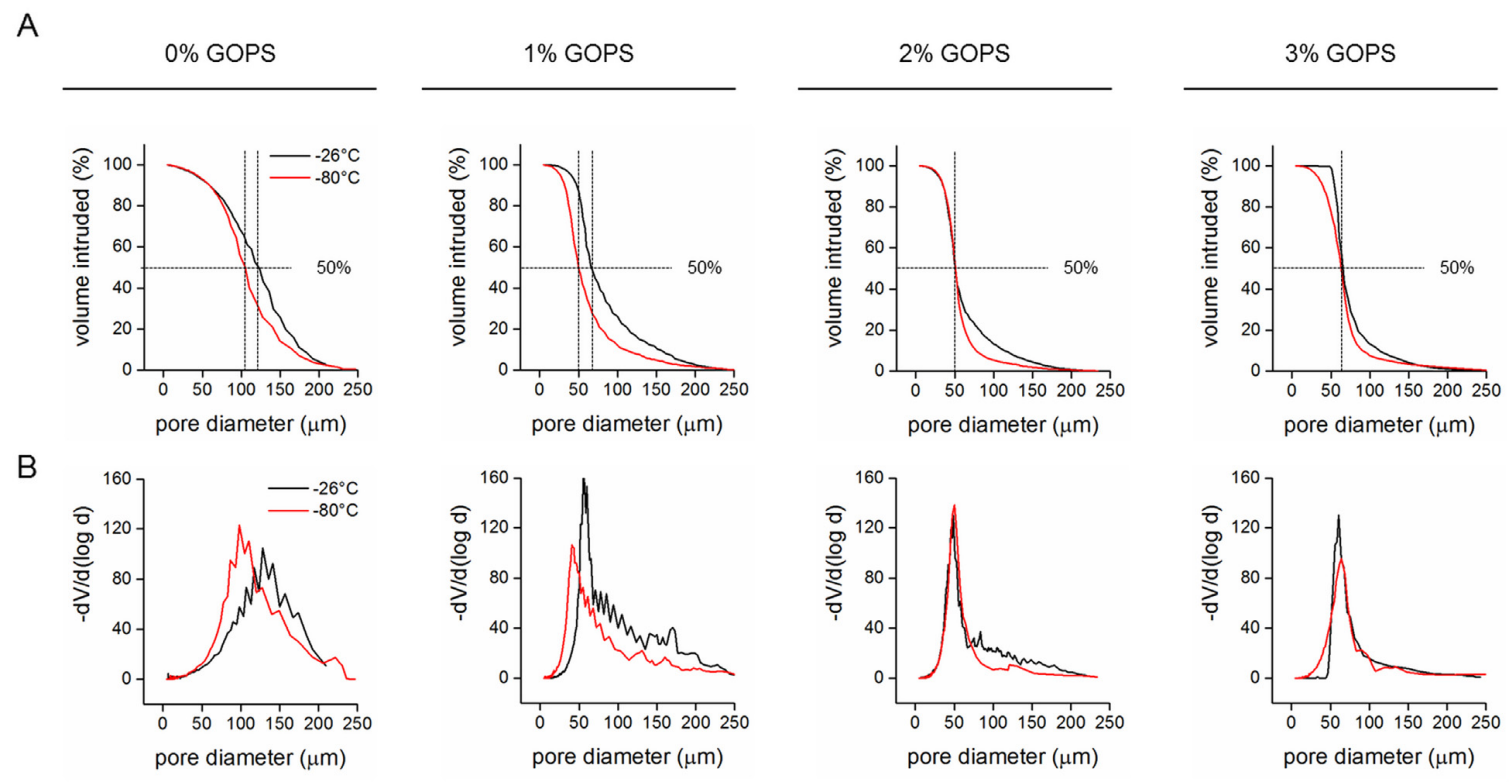
Representative curves obtained from mercury intrusion porosimetry for PEDOT:PSS scaffolds processed at different temperatures (−26 °C (black curve) or −80 °C (red curve)) and with different crosslinker concentrations (0, 1, 2, or 3 v/v% GOPS). A) Median pore diameter, at a mercury volume intrusion of 50% (dotted lines). B) Pore-diameter distribution. (For interpretation of the references to colour in this figure legend, the reader is referred to the web version of this article.)

Highly porous scaffolds were produced by freeze casting a PEDOT:PSS dispersion (Fig. 1). Based on earlier reports, we processed a 1.25 wt% PEDOT:PSS dispersion with crosslinker (3-glycidoxypropyltrimethoxysilane, GOPS) concentrations that were varied from 0 to 3 v/v% [13]. We additionally added a secondary dopant (4-Dodecylbenzenesulfonic acid, DBSA), at a constant concentration of 0.25 v/v%. PEDOT:PSS dispersions were frozen at a rate of −1 °C·min^−1^ when placed at -80 °C (according to the cellcool manufacturer’s specifications), whereas a slightly slower cooling rate was expected when placed at −26 °C. Pore interconnectivity (accessible pores) was evaluated by mercury porosimetry (Fig. 2). Both freezing temperature and crosslink concentration had an effect on median and mean pore diameter, with reduced pore diameters at reduced temperature and increasing GOPS concentration (Table 2). Mercury porosimetry during initial evaluation studies was only accomplished on a small sample selection, whereas the most reproducible results were found for scaffolds frozen at −26 °C with 2 v/v% GOPS addition. These scaffolds were characterised in depth and used for all further cell culture studies. Based on mercury volume intrusion measurements and using Eqs. (2), (3), scaffold pores exhibited a mean diameter d_mean_ = 13.7 ± 1.9 µm, a median diameter at 50% mercury volume intrusion of d_median_ = 53.6 ± 5.9 µm, and a total pore surface area of S = 7.72 ± 1.7 m^2^·g^−1^. Based on SEM measurements, median scaffold pore diameter was 93.7 ± 35.8 µm, laying well within the desired 100 µm pore size ideal for cell and nutrient infiltration. Pore diameters were assessed both on vertical and horizontal cross sections and were shown to be homogeneous throughout the scaffold.

**Table 2:** Median pore diameter of scaffolds produced at different temperatures and crosslinker concentrations. The highlighted scaffold (2% GOPS, −26 °C) was used for all further material characterisation and cell studies.

1 Based on mercury porosimetry, n = 1 for 0, 1, and 3 v/v% GOPS, n = 5 for 2 v/v% GOPS, processed at −26 °C.

2 Based on SEM images and analysis with ImageJ.

#### 3.1.2 Mechanical properties

**Fig. 3:**
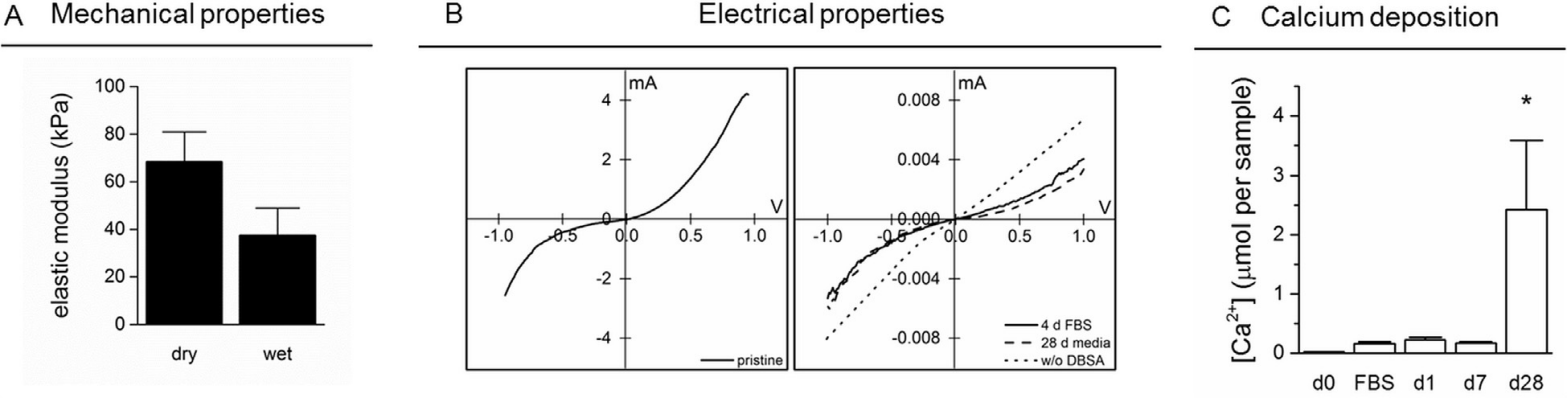
A) Mechanical properties of PEDOT:PSS scaffolds. Elastic modulus was assessed in the dry and wet (PBS incubated) state. B) I-V curves of scaffolds; measured directly after production or after incubation in FBS for 4 days or culture media for 28 days (scaffolds were all measured in the dry state). As a control, IV curves were recorded for scaffolds processed without the secondary dopant DBSA (dotted line). C) Calcium deposition on PEDOT:PSS scaffolds without cells. Ca^2+^ analysis was performed on as produced scaffolds (d 0), after incubation in FBS, and incubation in cell culture media for 1, 7 or 28 days to mirror cell studies. ^*^P < 0.05, compared to all other conditions.

Mechanical properties were assessed in the dry and wet state (incubation and measurements in PBS) and the elastic modulus was 68 ± 13 kPa and 37 ± 12 kPa, respectively (Fig. 3A). Of note, scaffolds maintained good mechanical and structural integrity throughout 28 days in cell culture media, confirming scaffold stability under physiological conditions. No major swelling or morphological changes were observed (results not presented).

#### 3.1.3 Electrical properties

Fig. 3B shows representative I-V curves at -1 to 1 V bias. Electronic conductivity was calculated based on the slope of the linear regression in the ohmic region. Electronic conductivity decreased after 4 days in serum but remained stable through 28 days of culture (σ = 1.4 ± 0.5·10^−4^, 2.0 ± 1.5·10^−5^, and 6.1 ± 4.0·10^−6^ S·cm^−1^, for day 0, day 4 or day 28, respectively). This reduced conductivity is thought to be associated with an initial burst release of the secondary dopant DBSA. As a control, the electrical conductivity of PEDOT:PSS scaffolds without DBSA was analysed based on I-V curves (Fig. 3B) and confirmed to lie in the same regime as serum incubated scaffolds (1.5 ± 0.5·10^−5^ S·cm^−1^). The electrical conductivity of the scaffolds does not significantly decrease after the initial reduction after 4 days, with a calculated conductivity of 6.1 ± 4.0·10^−6^ S·cm^−1^ after 28 days in cell culture media.

#### 3.1.4 Scaffold mineralisation

Further scaffold characterisation involved the analysis of calcium accumulation in cell culture media. Ca^2+^ analysis showed PEDOT:PSS alone induced calcium accumulation after 28 days (Fig. 3C). During the initial time points (day 1 and day 7) Ca^2+^ content is significantly lower than on day 28.

### 3.2 *In vitro* evaluation

**Fig. 4:**
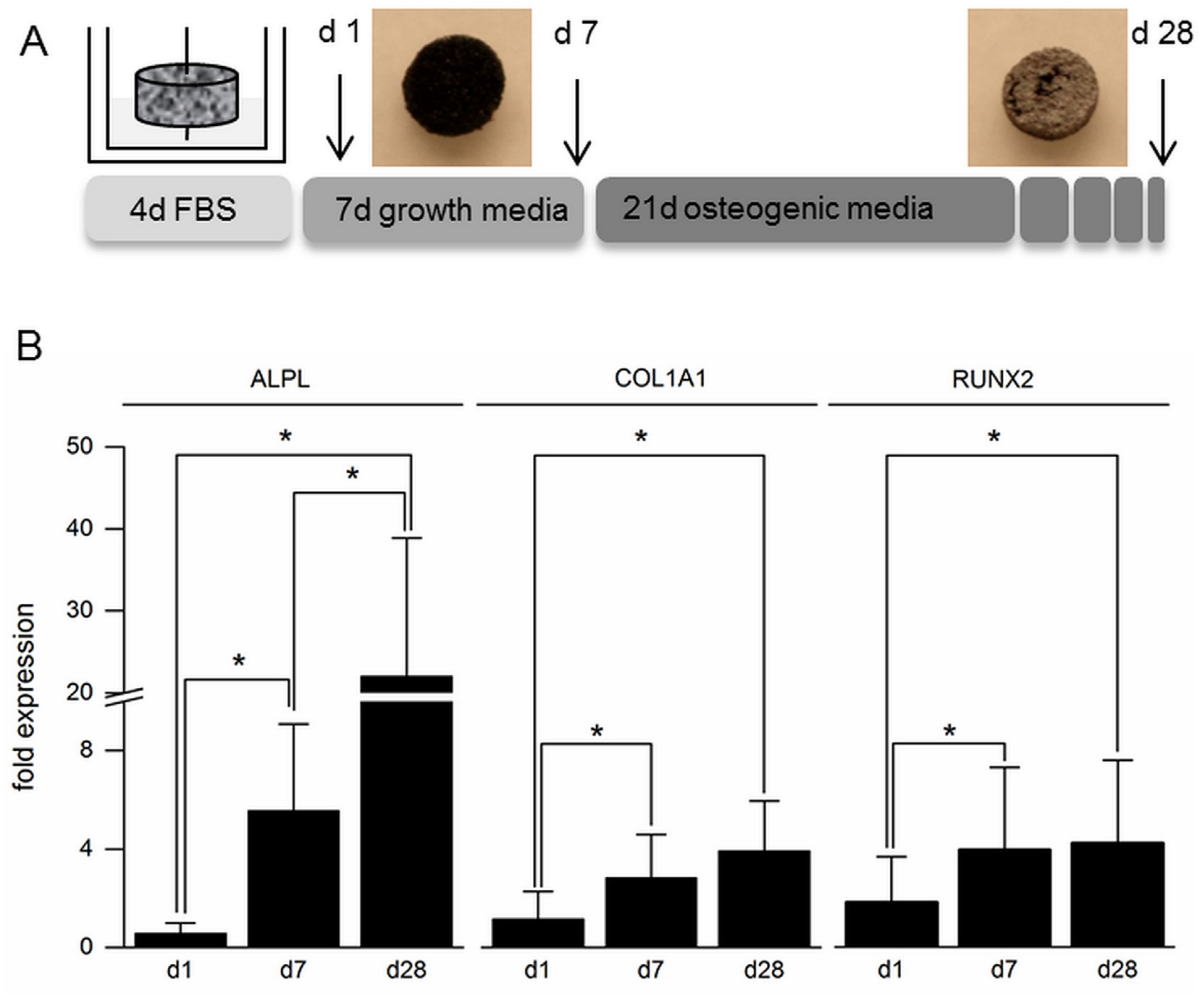
A) Schematic of experimental procedure. PEDOT:PSS scaffolds were placed in PDMS coated well plates, fixed with insect pins, and preconditioned in FBS for 4 days prior to cell seeding. MC3T3-E1 were cultured in growth media for 7 days, followed by differentiation in osteogenic media for 21 days. After a total culture period of 28 days, a white cell membrane was visible on the scaffolds by naked eye. B) Gene expression of osteogenesis related genes. qPCR was accomplished on day 1, 7 and 28 for *ALPL, COL1A1* and *RUNX2*. Gene expression was normalised to the house keeping gene 18S and to non-differentiated MC3T3-E1 on TCPS (day 0). ^*^P < 0.05, N = 4 with three replicates each, resulting in n = 12 scaffolds.

In the current study, MC3T3-E1 were seeded on PEDOT:PSS scaffolds and analysed at different time points, namely day 1 and day 7 post seeding (basal growth media) and day 28 (when scaffolds were switched to osteogenic media for the final 21 days). A time line of the experimental setup is displayed in Fig. 4A. qPCR of selected genes confirmed the commitment of MC3T3-E1 to the osteogenic lineage, with significantly enhanced expression of *ALPL, COL1A1* and *RUNX2* on day 28 compared to day 1 (Fig. 4B). All gene expression levels were normalised to the house keeping gene 18S and to MC3T3-E1 expanded on TCPS, prior to culture on PEDOT:PSS (day 0) according to the 2^−ΔΔCt^ method [31].

**Fig. 5:**
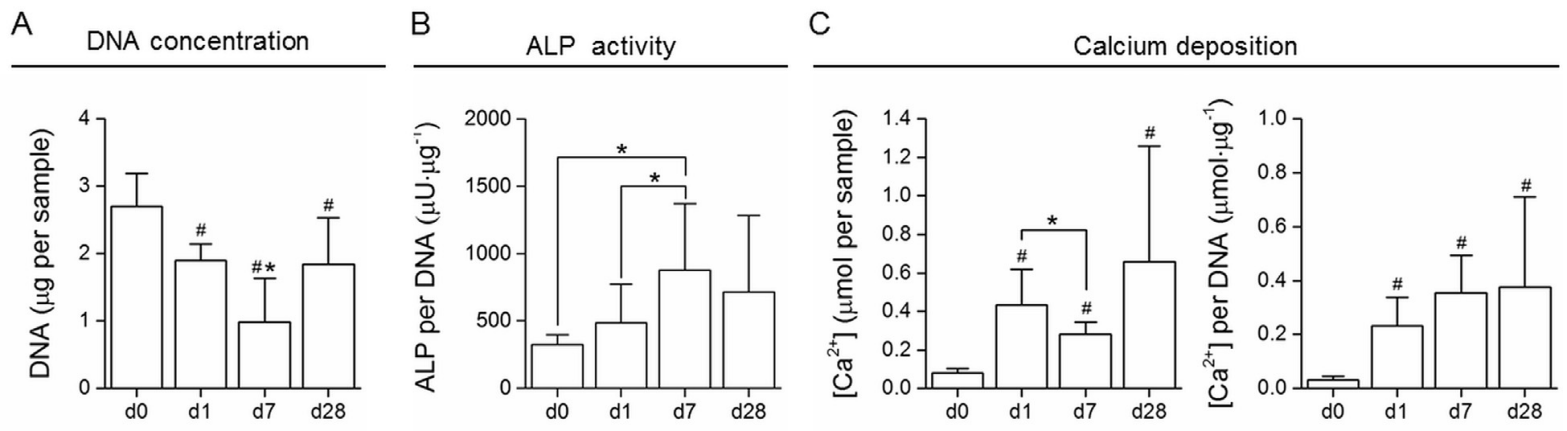
A) DNA quantification of MC3T3-E1 cultured on PEDOT:PSS scaffolds and lysed after 1, 7 or 28 days in culture (d0 refers to an aliquot of the initial cell seeding suspension). ^#^*P* < 0.05 compared to day 0, and ^*^P < 0.05 compared to all other time points. B) ALP enzymatic activity normalised to DNA. ^*^P < 0.05, where indicated. C) Calcium deposition of MC3T3-E1 during cell culture (per sample and normalised to DNA). A significantly enhanced Ca^2+^ concentration was found on day 1, day 7 and day 28 compared to day 0. ^#^*P* < 0.05 compared to day 0, and ^*^P < 0.05, where indicated. N = 4 with three replicates each, resulting in n = 12 scaffolds.

When comparing DNA values of day 1 to DNA values of day 0, the seeding efficiency on PEDOT:PSS scaffolds was calculated as being ∼73%, indicating good cell attachment and retention after 24 hours. DNA quantification revealed a decrease in cell number after 7 days and an increase to a cell number comparable to day 1 after an additional 21 days in culture (Fig. 5A). A decrease in cell number during an initial phase is a common phenomenon on scaffolds, which can be attributed to a relatively weak cell attachment to the synthetic material. Over time, cells deposit their own ECM, producing an enhanced biointerface and allowing for further cell proliferation, attachment, and overall an increased number of cells. Enzymatic activity of alkaline phosphatase (ALP) was recorded at similar time points and normalised to DNA content (Fig. 5B). There was no significant increase in ALP activity per DNA when comparing day 28 to all other time points, whereas on day 7, at the onset of osteogenic differentiation, a significantly higher ALP activity compared to day 1 and day 0 was found. Ca^2+^ deposition, indicative for ECM mineralisation, increased with ongoing culture under differentiation conditions and was significantly enhanced on day 28 compared to day 0 (Fig. 5C).

**Fig. 6:**
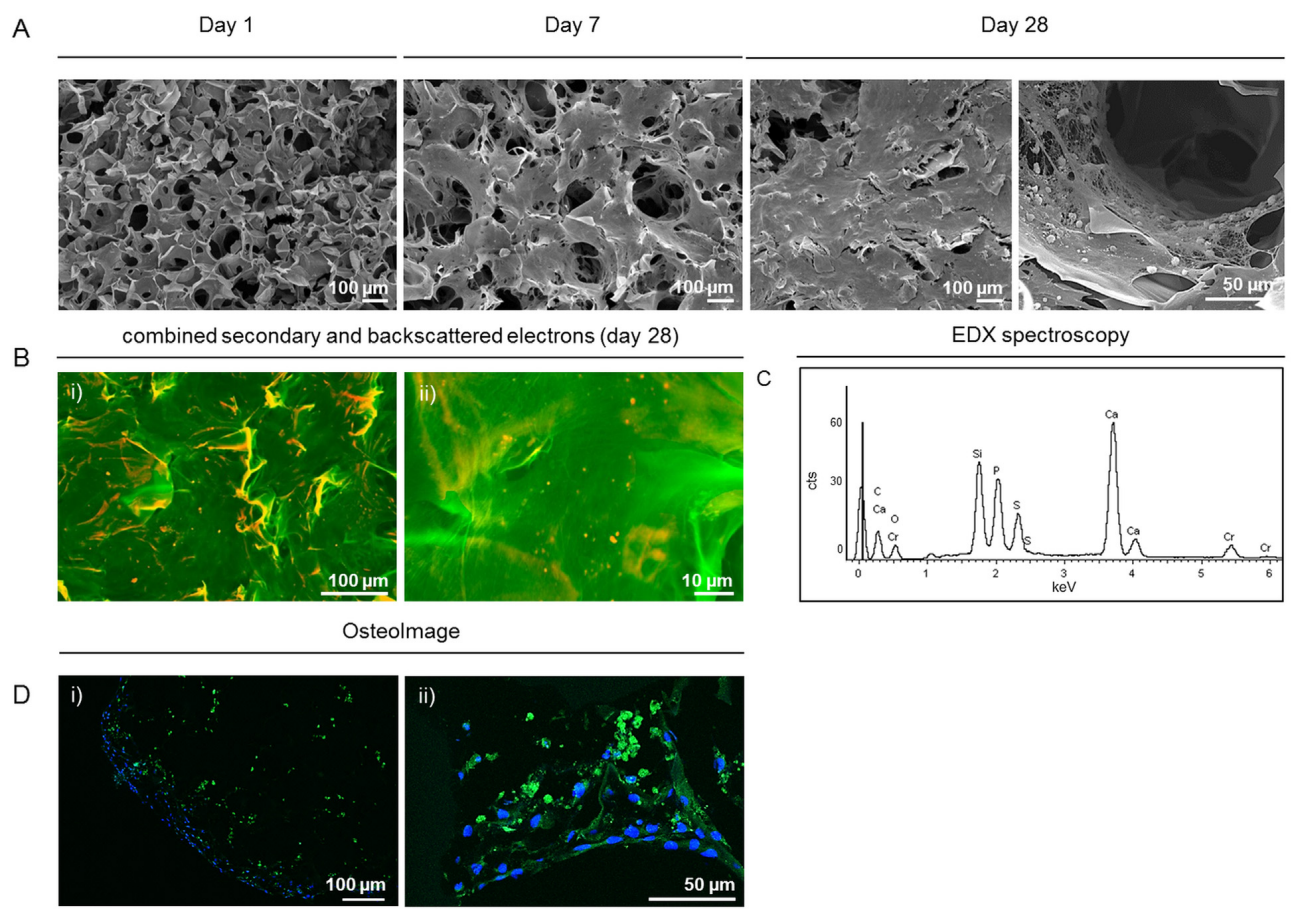
A) SEM images of MC3T3-E1 cultured on PEDOT:PSS scaffolds. Samples were harvested on day 1, 7 and 28 post seeding. SEM images show an increased cell number and matrix deposition over time, with a densely formed tissue-construct, containing bone nodules after 28 days in differentiation media. B) i) and ii) False coloured SEM image of backscattered and secondary electrons. Calcified particles are presented in red, organic material in green. C) EDX spectrum of MC3T3-E1 after 28 days under osteogenic culture conditions. The spectrum shows peaks of high intensity for Ca and P, main components of hydroxyapatite Ca_10_(PO_4_)_6_(OH)_2_. SEM image acquisition and EDX analysis were performed on the surface of the scaffolds, at randomly located spots. Homogeneous coverage was observed for all time points. D) i) and ii) Confocal microscopy images of OsteoImage stained MC3T3-E1 on day 28. Mineralised bone nodules appear green, while nuclei are counterstained with DAPI (blue). Images were acquired on cryosections, sliced from the centre of the scaffolds after an initial transversal cut and consecutive embedding.

SEM images, acquired at the same time points, indicate a continuously increasing cell number and matrix deposition after prolonged time in culture compared to day 1 (Fig. 6A). A very dense ECM layer, covering the entire scaffold surface is apparent by day 28. PEDOT:PSS pores and scaffold structures cannot be distinguished, and a tissue-like structure has been formed. An advanced SEM imaging method of combined backscattered and secondary electron collection that we developed was used to distinguish mineralised particles within the deposited organic extracellular matrix [32], and EDX spectroscopy was used to characterise the nodules seen on standard SEM images. The increased brightness of the particles displayed in Fig. 6B indicates the abundance of inorganic structures of higher density, characteristic for mineral particles. Specific peaks in the EDX spectra at 0.3, 3.7 and 4 keV, and 2 keV further confirm the presence of calcium and phosphate, respectively. A hydroxyapatite specific staining (OsteoImage) was used to further characterise the mineral formation within the matrix [33]. Fig. 6D shows confocal microscopy images of positively stained bone nodules. The specific staining distinguishes hydroxyapatite nodules from other calcium crystals. These nodules were not found on images of earlier time points and no positive staining was observed on day 1 or day 7.

Combining the methods of Ca^2+^ quantification, SEM imaging, and hydroxyapatite staining, we could confirm the formation of mineralised, extracellular matrix. This suggests osteogenic lineage commitment of MC3T3-E1 on PEDOT:PSS scaffolds and the differentiation into a more mature phenotype.

**Fig. 7:**
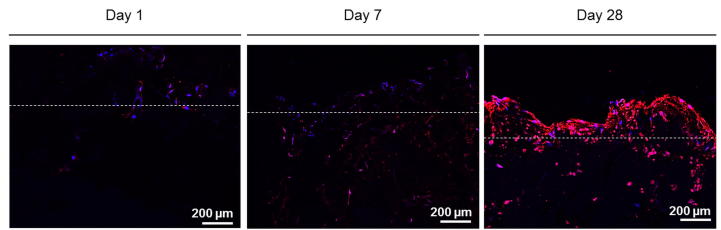
Confocal microscopy image of osteocalcin positively stained MC3T3-E1 (red, nuclei counterstained in blue) at different time points. Dotted lines give a reference for the scaffold border. Images were acquired on cryosections, sliced from the centre of the scaffolds after an initial transversal cut and consecutive embedding.

Further evidence for osteogenic matrix formation was provided by osteocalcin stainings and confocal image acquisition. Fluorescent images of osteocalcin positively stained cross sections are displayed in Fig. 7. After 1 or 7 days in culture, osteocalcin was present at a low concentration and as supported by SEM imaging, only limited ECM formation was observed. After 28 days in culture, a dense cell multilayer has formed on the surface of the scaffold.

## 4 Discussion

We could demonstrate the development of a highly porous, conductive scaffold based on PEDOT:PSS that supported the differentiation of pre-osteogenic precursor cells (MC3T3-E1) into mature osteoblasts.

Generating scaffolds of high pore interconnectivity and pore diameters above 100 µm has been of major interest in many tissue engineering approaches and has been reported to be of importance for cell fate, infiltration and three dimensional tissue formation [34], [35]. Among other techniques, ice templating, or freeze casting, has been increasingly considered to process various polymers of synthetic or natural origin [36], [37], [38]. This method is particularly interesting for conjugated polymers, since it allows to process inherently insoluble polymers in an aqueous dispersion. Pore size diameter and scaffold morphology can be controlled by adjusting solvent weight ratio, freezing temperature, freezing velocity, and crosslinker concentration. Here we optimised pore sizes by varying the freezing temperature and crosslinker concentration. The obtained results lie well in agreement with earlier results published by Wan *et al.* [13] who reported on reduced pore diameter at increasing GOPS concentration, freezing rate, and PEDOT:PSS wt%. Further, the median pore diameter of ∼50 µm is in line, if not surpassing, earlier reported results of pore diameters of 39.1 ± 2.7 µm for scaffolds processed under comparable conditions [13]. Although we found a similar trend of reducing pore size with adjusted parameters using different methods, we also found a discrepancy in pore sizes between SEM and mercury porosimetry readings. Porosimetry relies on mercury intrusion, which is strictly limited to accessible pores and gives a measure for the scaffold’s pore interconnectivity, whereas image analysis is purely based on the large open pore structure at the imaged cross section and neglects the interconnectivity of pores, mainly formed by hourglass shaped structures. In addition, soft, porous scaffolds undergo a significant compression during mercury intrusion, resulting in underestimated pore diameter [39]. There is currently no consensus on the ideal pore diameter for bone tissue engineering and conflicting results between 85 to 325 µm are reported. However, there is a general agreement that pore sizes above 100 µm in diameter would allow for enhanced cell attachment and fate [40], [41].

These highly porous scaffolds displayed good mechanical integrity under physiological conditions and the compression load experiments provide evidence that the requirements for stable mechanical integrity *in vivo* are also met with the current scaffold design. The observed lower elastic moduli for scaffolds in aqueous environments are a common phenomenon and often reported for both hydrophilic and hydrophobic polymers [42]. The decrease in mechanical properties can be explained by a wetting process and resulting water absorbance (without observed swelling) and thus a plasticisation of the polymer that leads to a decreased Tg and lower elastic modulus. Bone, appearing in its many forms and executing distinct functions from load bearing to organ protection, also has unique mechanical properties, ranging from 0.1 to 1 GPa for cancellous bone and around 10 GPa for cortical bone. These properties could only be matched by artificial hydroxyapatite scaffolds or ceramics [17]. Elastic moduli of several GPa reflect mechanical properties of bulk native bone. However, cells are sensitive to their micro-environmental elasticity and the commitment to an osteogenic state has been demonstrated on substrates in the range of several kPa [43], which this scaffold provides.

PEDOT:PSS substrates, in the form of thin films, have primarily found interest in the fields of biosensors, photovoltaic and optoelectronics, where electrical properties, thermal stability, and crystallinity have been well characterised [4], [44], [45], [46]. Electrical conductivity and stability of three dimensional scaffolds in physiological environments has been addressed in less detail. Indeed, the material properties of three dimensional PEDOT:PSS scaffolds in the dry state, without the addition of electrolytes, or after prolonged incubation in cell culture media, have not been characterised in previous studies, to the best of our knowledge. The conductivity of PEDOT:PSS films or substrates has furthermore been reported to be highly process dependent, in particular varying depending on added solvents, annealing temperature or material morphology [47], [48], [49]. It is therefore not surprising that the 3D scaffolds presented herein exhibit a lower conductivity than usually reported for 2 D films (10^−3^ S·cm^−1^ up to 10^2^ S·cm^−1^). DBSA was used as a secondary dopant based on previous studies that reported enhanced conductivity of DBSA doped material and facilitated processing and casting of PEDOT:PSS films [11], [50]. An initially high conductivity after DBSA addition was observed, with a reduced, yet stable conductivity after prolonged incubation in media. These long term incubated scaffolds are still more conducting than typical tissue engineering scaffold materials. Of note, the high porosity of the scaffolds and the pronounced amount of void space results in an overestimation of the accessible surface area and actual thickness of the material for electronic transport. This adds an additional challenge for direct comparison to 2D films. The high porosity and structured surface furthermore hampers scaffold electrode connection as opposed to 2D films and the contact area scaffold-electrode is significantly reduced due to the non-planar surface of the sample. The sigmoidal behavior shows that there is no pure ohmic contact between the electrodes and the scaffold, indicative of an energetic injection barrier. In this respect, the current electrical evaluation presents several limitations that need to be taken into account when comparing conductivity values of highly porous 3D structures to 2D films. Importantly, however, these errors remain the same for all evaluated scaffolds and comparison between pristine, FBS or medium incubated scaffolds remains valid. With these measurements we wish to emphasise that the DC electrical properties of the scaffold remain unaffected after the initial loss of conductivity.

Despite research efforts in several fields of tissue engineering, there is no consensus on the cues that conductive materials provide to cells. The running hypothesis is that conductive substrates would allow for direct stimulation of cells to increase differentiation. External stimulation of bone cells has been shown to increase matrix mineralisation, yet no increased gene expression of osteogenic markers was reported [23], [24], [25], [26]. Our results provide evidence that PEDOT:PSS scaffolds can be used as substrates for bone tissue engineering, yet further investigations are needed to assess the whole potential of the herewith designed scaffolds and to understand underlying mechanisms.

During osteogenic differentiation, cells go through different stages and reach a mature phenotype upon extracellular matrix mineralisation and hydroxyapatite bone nodule formation. Scaffold mineralisation as a form of material functionalisation strategy can act as a vital support to initiate nucleation and further trigger osteogenic differentiation and ECM mineralisation [51], [52], [53]. Hybrid scaffolds of PEDOT:PSS, containing bioactive glasses and gelatine have been shown to mineralise after prolonged incubation in simulated body fluid (SBF) and were reported to be potent materials to induce biomineralisation *in vitro* [54]. Mineralisation of biomaterials has commonly been assessed by incubation in SBF over several days or weeks. In particular, electrospun fibres of poly(lactic acid) or poly(caprolactone) were shown to present crystalline depositions of calcium phosphates and hydroxyapatites [55], [56]. To our knowledge, mineralisation of PEDOT:PSS alone has not been demonstrated previously and the extent to which PEDOT:PSS alone can initiate cell mineralisation remained unresolved. Nucleation of mineralisation or hydroxyapatite formation is thought to be triggered with an initial accumulation of calcium. The ability to accumulate calcium as observed here is a promising step towards possible scaffold mineralisation [28]. This calcium deposition after incubation in serum or media is expected to support crystal nucleation and ECM mineralisation of osteoblast precursor cells.

Interestingly, we found higher Ca^2+^ concentration on cell-free scaffolds compared to cell-seeded scaffolds (on day 28). We are attributing these findings to the excessive matrix deposition of MC3T3-E1 after this prolonged culture period and the resulting difficulties to dissolve and quantify calcium deposition. On cell-free scaffold, however, dissolution of salts in acid is a straight forward step which could potentially result in an increase in the detected amount. The absolute amount of Ca^2+^ per sample drops significantly after 7 days, which is directly correlated with a drop in cell number, assessed by DNA quantification. Normalised to DNA, however, we did not see this drop on day 7, but observed a significant increase in Ca^2+^ accumulation compared to day 0, whereas no significant differences were observed when comparing day 28 to day 7 or day 1. Interestingly, it has been reported that the mineralisation potential of MC3T3-E1 decreases with increasing passage number and highly depends on media formulations [57], which indicates some challenges with respect to mineralisation of this cell line. Despite experimental repeats with MC3T3-E1 cells within the same passage numbers and similar media formulations, these potentially occurring variations in mineralisation might be reflected in the high standard deviations observed here, leading to non-significant differences when comparing day 28 to day 7. However, values of 20 to 60 µg·mL^−1^ reported in the publication by Yan *et al.* [57] lie well in agreement with the herewith obtained value of 0.7 µmol per sample on day 28 (corresponding to 56 µg·mL^−1^). Furthermore, our results on OsteoImage staining, SEM imaging, and EDX spectroscopy after 28 days further support matrix mineralisation. Taken together, these results point towards osteogenic differentiation of MC3T3-E1 precursor cells. It remains open whether further stimuli, for instance external electrical stimulation, would increase calcium accumulation, as reported in earlier studies with SaOS-2 osteoblast-like cells or rat bone marrow stromal cells [23], [24], [26]. MC3T3-E1 osteogenic precursor cells are a commonly used cell line for osteogenic tissue engineering with similar differentiation and maturation steps as native osteoblasts [58]. Their differentiation into osteogenic cells can be monitored in a variety of ways, such as their ALP activity, gene expression or ECM mineralisation. For scaffold evaluation, these cells form a versatile model system and provide relevant information prior to further assessment. Further studies would shed light on whether mesenchymal stem cells or primary osteoblasts would show a different response on PEDOT:PSS scaffolds and develop a mature osteogenic phenotype of pronounced mineralisation.

The results on gene expression suggest that MC3T3-E1 are in an early phase of osteogenic differentiation. *ALPL* is a transiently expressed gene with a peak at the onset of differentiation, while *RUNX2* is a marker for early osteogenic differentiation. It is therefore expected that gene expression of *ALPL* would drop at later time points, whereas *RUNX2* and *COL1A1* will further increase. Of note, gene expression was significantly increased on day 7 compared to day 1. At this time point, MC3T3-E1 cells were cultured in basal media only. This suggests that osteogenic precursor cells spontaneously differentiate into osteoblasts on our scaffolds, indicative that PEDOT:PSS supports osteoinduction. The prolonged culture period in osteogenic media further drove MC3T3-E1 into more mature osteoblasts with increased gene expression levels.

Based on these results, we suggest that cells proliferate over time, form a densely packed multilayer with enhanced mineralised ECM deposition, and differentiate into mature osteoblasts. The current study provides evidence that PEDOT:PSS is a suitable scaffold for bone tissue engineering and further research, using stem cells and/or electrical field stimulation is warranted. In the interest of animal welfare and ethical reasons, scaffold evaluation in established *in vitro* model systems is crucial to any further *in vivo* studies and our results provide pivotal steps for further investigations.

## 5 Conclusion

Highly porous, conductive scaffolds were produced by freeze drying a PEDOT:PSS dispersion. Scaffolds present high pore interconnectivity and a median pore diameter above 50 µm, allowing for cell infiltration and matrix deposition within the void space. We could demonstrate that PEDOT:PSS is suitable as a scaffold for bone tissue engineering, indicated by the differentiation of osteogenic precursor cells (MC3T3-E1) into osteocalcin positively stained osteoblasts that express significantly enhanced levels of *ALPL, RUNX2* and *COL1A1* and deposit mineralised ECM.
